# MELD-Na Alterations on the Liver Transplant Waiting List and Their Impact on Listing Outcome

**DOI:** 10.3390/jcm12113763

**Published:** 2023-05-30

**Authors:** Gerd R. Silberhumer, Georg Györi, Jonas Brugger, Lukas Baumann, Sonja Zehetmayer, Thomas Soliman, Gabriela Berlakovich

**Affiliations:** 1 Department of Transplant Surgery, Medical University of Vienna, 1090 Vienna, Austria; 2 Department of CeMSIIS, Center for Medical Statistics, Informatics and Intelligent Systems, Medical University of Vienna, 1090 Vienna, Austria

**Keywords:** liver allocation, liver transplant waiting list, MELD score alterations, Delta MELD

## Abstract

Background: Dynamic MELD deterioration (Delta MELD) during waiting time was shown to have significant impact on post-transplant survival. The aim of this study was to analyze the impact of MELD-Na score alterations on waiting list outcomes in liver transplant candidates. Method: 36,806 patients listed at UNOS for liver transplantation in 2011–2015 were analyzed according to their delisting reasons. Several different MELD-Na alterations during waiting time were analyzed (e.g., maximal change, last change before delisting/transplantation). Outcome estimates were calculated according to MELD-Na scores at listing and Delta MELD. Results: Patients who died while on the waiting list showed a significantly higher deterioration in MELD-Na during the waiting time (6.8 ± 8.4 points) than stable patients who remained actively listed (−0.1 ± 5.2 points; *p* < 0.01). Patients who were considered too healthy for transplantation improved by more than 3 points on average during the waiting time. The mean peak MELD-Na alteration during the waiting time was 10.0 ± 7.6 for patients who died on the waiting list, compared to 6.6 ± 6.1 in the group of patients who finally underwent transplantation. Conclusions: Deterioration of MELD-Na during waiting time and maximal MELD-Na deterioration have a significant negative impact on the liver transplant waiting list outcome.

## 1. Background

The MELD score is established worldwide as a means of ranking patients on the waiting list for liver transplantation [1]. This objective tool, based solely on reliable laboratory scores rather than subjective classification of clinical appearance, allows listing potential liver transplant recipients according to their disease severity and their need for a new organ [2]. MELD score allocation significantly reduces waiting list mortality, as the next available organ is allocated to the patient with the most urgent need. Nevertheless, experts still discuss this “sickest first” policy and it remains controversial due to its impact on post-transplant outcome [3]. 

Discussion is ongoing that the MELD score does not perfectly reflect the clinical situation of liver transplant candidates [4]. Several refinements have been published to cover these requests but they have not gained clinical relevance. Nevertheless, the very initial postulation of adding Na to the MELD formula resulted in the establishment of MELD-Na as a listing tool in the US [5,6]. Recent studies have proven that the addition of Na reduced waiting list mortality, especially in hyponatremic patients [6,7]. Nevertheless, to date, a slightly worsened post-transplant outcome has been reported for unclear reasons [7].

Previously published data from Eurotransplant showed that dynamic alterations of the MELD score (Delta MELD) have a significant impact on post-transplant survival. In particular, patients with an MELD deterioration of more than 10 points during waiting time face significantly poorer post-transplant outcomes [8,9].

Limited data is available on waiting list outcomes of patients according to their dynamic MELD-Na changes during waiting time. 

The aim of this study was to analyze various MELD-Na score alterations during waiting time and their impact on waiting list outcomes.

## 2. Methods

**Figure 1:**
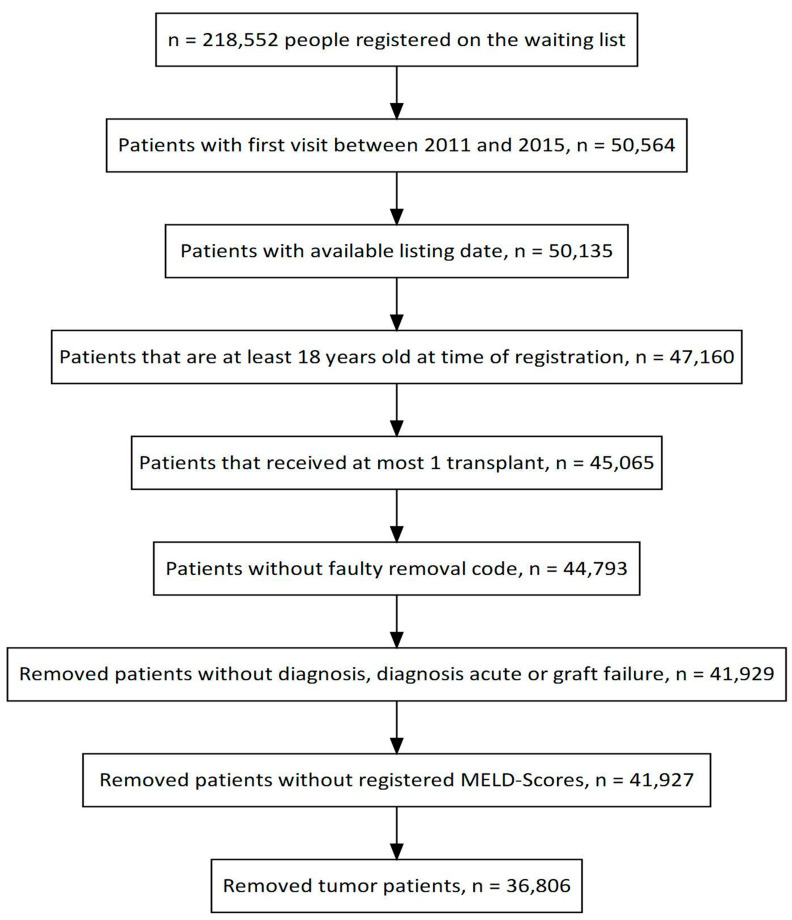
Flowchart for selection of patients.

According to the UNOS database, more than 50,000 patients were listed for liver transplantation for the first time between 2011 to 2015. Patients listed for re-transplantation and patients below 18 years of age were excluded from analysis. Laboratory MELD-Na values were exclusively considered for analysis, as weighing of standard exceptions and consecutively related prioritization of these patients on the waiting list were not the topics of this study. Consequently, patients listed for acute hepatic failure were excluded from analysis, as were patients listed for multi-organ transplantation. Further, patients listed for HCC were excluded from analysis as MELD-Na does not reflect HCC prioritization. A flowchart illustrates the final study cohort of 36,806 patients (Figure 1).

Patients were classified according to the following listing indications: alcoholic, biliary, chronic hepatitis, metabolic cirrhosis, NASH, and others.

### 2.1. MELD Score Calculations

The MELD score was calculated according to the following formula: Refs. [1,10]

MELD = [0.957 × ln(creatinine mg/dL) + 0.378 × ln(bilirubin mg/dL) + 1.12 × ln(INR) + 0.643] × 10

The MELD-Na score was calculated according to this formula: Ref. [5]

MELD-Na = [0.025 × MELD × (140 − Na)] + 140 (Na ranges from 125 to 140)

Waiting time was defined as the period between active listing to delisting (for still actively listed patients, the last available MELD score measurement). 

MELD On was defined as the MELD score at the time of listing.

MELD Off was defined as the last available MELD score at the time of delisting.

Delta MELD was calculated as the difference between MELD Off and MELD On.

Delta MELD max was defined as the maximal difference between the lowest and highest MELD measurements during the waiting time.

Delta MELD last was calculated as the difference between the delisting or last available MELD calculation (MELD Off) and the next-to-last available MELD score. 

Delta MELD “last 30” was calculated as the difference between MELD off and the MELD score 30 days before MELD off or earlier. 

The same calculations were applied for MELD-Na. Differences in outcome between MELD and MELD-Na were compared. 

Patients were analyzed according to their waiting list outcome (delisting reasons), which were defined as having undergone transplantation (“TX”), for other reason (“other”), died on list (“DOL”), removed due to recovery (“too good”), removed due to medical deterioration (“too poor”), or still actively listed (“active”). Waiting times depending on waiting list outcome and MELD-Na assessments were analyzed. 

Further, subgroup analyses of patients listed less than 30 days were performed.

### 2.2. Statistics

Arithmetic mean and standard deviations were calculated for all MELD-Na measurements (MELD-Na On, MELD-Na Off, Delta MELD-Na, Delta MELD-Na max, Delta MELD-Na last, and Delta MELD-Na “last 30”). Median and first and third quartiles are reported for waiting time parameters (total waiting period, time between MELD-Na assessments, time between the last two MELD-Na assessments). The same summary statistics were computed separately for patients listed for less than 30 days. 

All results were obtained for both MELD and MELD-Na. All calculations were performed with regards to listing indication and delisting reasons.

The Kruskall–Wallis test was performed to test for differences in waiting time with regards to listing indications, outcome classifications, and MELD-Na On, summarized in five categories. As a post hoc analysis, Wilcoxon rank-sum tests were performed to evaluate differences in waiting time between the TX group (reference group) and other outcome classifications. To test whether MELD-Na scores differed between outcome classifications, *t*-tests were performed using the TX group as the reference category. Differences in MELD-Na scores regarding listing indications were evaluated using ANOVA. 

Two competing risk regressions by Fine and Gray were computed for each competing event (DOL, Other, Too Good, Too Sick, TX)—the first one included MELD-Na On as an independent variable. Probabilities for each event occurring within 30 days were computed for MELD-Na On intervals of 0–14, 15–20, 21–29, 30–34, and 35 or more. 

The second model estimated probabilities for events within 30 days from the first time a patient reached an increase in MELD-Na score of 2–3, 4–5, 6–7, 8–10, and >10, compared to MELD-Na On. Cumulative incidence curves were drawn for each MELD-Na On and MELD-Na increase category and event. 

No correction for multiple testing was applied, therefore all *p*-values were of descriptive, hypothesis-generating character. Statistical analysis was performed using R version 3.6.1 or higher.

## 3. Results

### 3.1. Waiting List Outcome

Half of the patients listed (48.1%) had already undergone transplantation (TX group), 19.7% were still actively listed, 11.1% were removed due to poor medical condition, 12.0% died on the waiting list, 2.9% were removed due to recovery, and 6.2% of patients were removed for other reasons. Details are shown in Table 1.

We further analyzed the distribution of indications for transplantation according to the outcome classifications. The listing indications showed stable rates during the study period except for a decrease in the number of patients with hepatitis in favor of an increase in the number of patients with NASH. 

### 3.2. Waiting Time

The median waiting time for patients who underwent transplantation was 90 days (16:260) in this study population. DOL patients or those considered in too poor health for transplantation showed comparable median waiting time periods (DOL: 123 (28:344); too poor: 144 (34:363)). Patients who were considered in too good health for transplantation had a median waiting time of 531 days (277:868). 

Patients with the highest listing MELD-Na scores (MELD-Na On) experienced the shortest periods on the waiting list. The median waiting time periods were comparable between listing groups, except for those patients suffering from metabolic cirrhosis (90 for metabolic vs 147–192 days for all other classifications). 

Further details according to waiting time are shown in Table 2.

### 3.3. MELD-Na Assessments

The median MELD-Na assessment for all patients was 5 (2:9). 

Patient who died on the waiting list (median 5; 3:10), who were considered too sick (median 5; 3:10), and those who were still actively listed (median 5; 3:12) had comparable measurements. Patients who had already undergone transplantation had a median MELD-Na assessment of 4 (2:8).

The median time between MELD-Na assessments was 43 days (12:90). Patients who died on the waiting list showed a significantly longer median time period between assessments than patients who finally underwent transplantation ((28 (9:64) for DOL and 24 (6:66) days for TX; *p* < 0.01)). Patients who were considered in too good health for transplantation showed a median time frame of 107 (73:204) days between measurements. The median intervals between the last two MELD-Na assessments were significantly different for the TX and DOL groups ((8 (3:45) vs. 14 (4:54) days; *p* < 0.01)). Longer intervals were observed for patients in too good health for transplantation and those still waiting for transplantation ((median 127 (71:285) and 88 (38:130) days)). 

The median intervals between the timepoints of the MELD-Na max and last assessments were 0 days for DOL patients, 2 days for too sick patients, and 4 days for patients who underwent transplantation. Patients removed from the waiting list for being in too good medical condition presented with their MELD-Na max around one year before delisting (median). 

### 3.4. Meld-Na Values

The extension to MELD-Na did not have a significant impact on outcome prediction in this study population compared to MELD alone. (*p* = ns). 

MELD-Na values according to the outcome on the waiting list are shown in Table 3.

MELD-Na On was significantly higher for patients who died on the waiting list than for patients who finally underwent transplantation (*p* < 0.01). Patients removed from the waiting list due to too poor clinical condition had MELD-Na On values comparable to those of patients who underwent transplantation (*p* = 0.2).

MELD-Na Off was significantly higher for DOL patients and those who were removed due to poor medical condition than for those who finally underwent transplantation or patients classified as being too healthy for transplantation (both *p* < 0.01). 

DOL patients showed a mean Delta MELD-Na of 6.8 ± 8.4. Patients who were still awaiting a graft showed almost no Delta MELD-Na (−0.1 ± 5.2), and patients who were considered too healthy for transplantation improved by more than 3 points. 

Delta MELD-Na max was notably higher in the DOL and too poor groups than in the transplanted group (*p* < 0.01). 

Delta MELD-Na last for patients who died on the waiting list showed a mean deterioration of 2.8 ± 6.4 points, and patients considered to be too sick for transplantation showed a mean deterioration of 2.0 ± 5.7. The other groups showed stable MELD-Na scores at the last two MELD-Na assessments. 

Delta MELD-Na “last 30” showed characteristics comparable to MELD-Na last, with doubled values for DOL patients and almost doubled values for candidates rated in too poor health for transplantation, compared to those patients who underwent transplantation.

**Figure 2:**
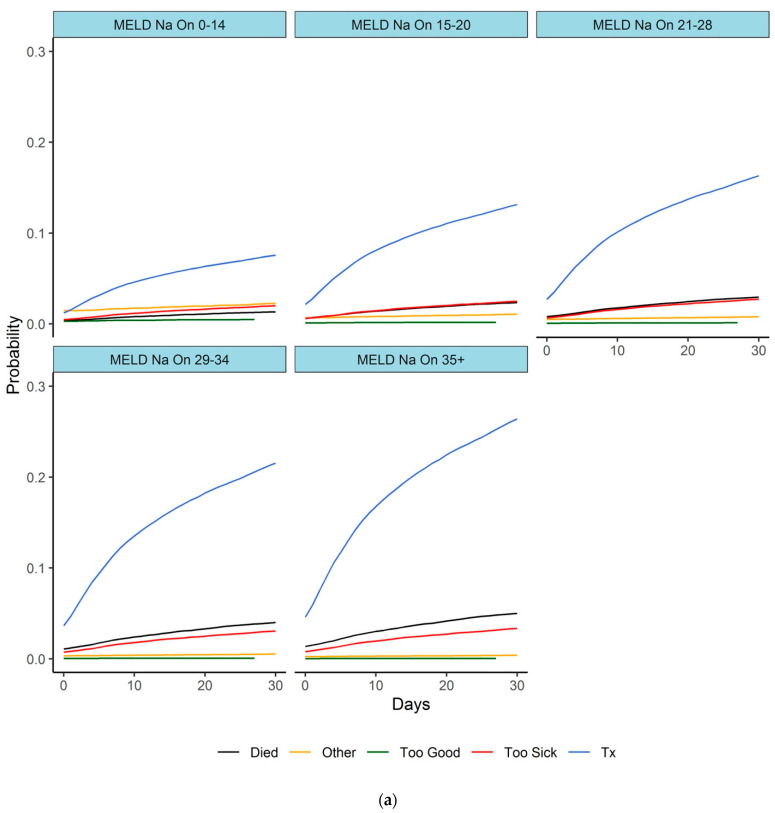

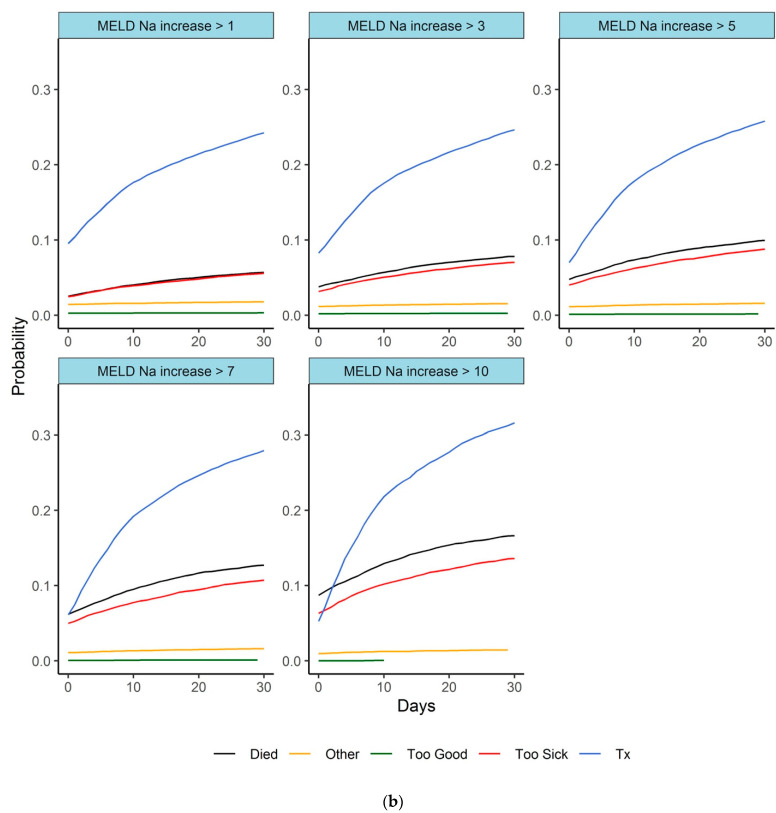
(**a**) Outcome scenarios within 30 days depending on MELD-Na On. (**b**) Outcome assessments for the next 30 days were estimated for Delta MELD-Na groups. Competing risk regression by Fine and Gray for MELD-Na On (**a**) and Delta MELD-Na (**b**). (**a**) Patients are grouped in MELD-Na On intervals 0–14, 15–20, 21–29, 30–34, and 35 or more. The Y-axis states the probability of an event (Died on list, Transplantation, Removed too good, Removed too poor, Other), while the X-axis shows the time in days. (**b**) Patients are grouped according to a “first MELD-Na increase” by more than 1, 3, 5, 7, and 10 points. Patient groups are not disjointed; therefore, patients can be included in more than one group.

Depending on a given MELD-Na On, we estimated various risk scenarios for the first 30 days on the waiting list, which are shown in Figure 2a. Similar probabilities of an event occurring within the next 30 days were calculated depending on different “first time increases in Delta MELD-Na (increase by more than 1, 3, 5, 7, or 10 points). Details are shown in Figure 2b. Patients with a higher MELD-Na On measurement were at higher risk of an unfavorable outcome; e.g., the probability of death within 30 days was 1.3% for patients with MELD-Na On <15 and increased to 5% for patients with MELD-Na On >35. Analogously, the probability of death within 30 days for patients with a Delta MELD-Na between 1 and 3 was 5.7%, and it increased to 16.7% for patients with a Delta MELD-Na >10.

### 3.5. Subgroup Short Waiting Time (<30 Days)

Almost one quarter of patients (24.2%) were delisted within 30 days or less. Their mean MELD-Na On was 27.5 ± 10.0 and their mean MELD-Na Off was 28.5 ± 10.4 points. The mean Delta MELD-Na max was 3.1 ± 4.0, and the mean Delta last was −0.4 ± 3.8. These patients had a median of two MELD-Na calculations. Sixty-four percent of these patients underwent transplantation, 12% died while on the waiting list, and 11% were removed due to having too poor medical condition. Further details are shown in Table 4.

### 3.6. Indications and MELD-Na Dynamics

Patients listed for metabolic disease (90; 10:310 days) showed a significantly shorter median waiting time than those with other listing indications (medians ranging from 147 to 192 days; *p* < 0.01). 

Delta MELD-Na increases ranged from a mean of 1.7 ± 6.8 for alcoholic patients to 3.8 ± 7.0 points for NASH patients. Mean Delta Max ranged from 6.8 ± 6.4 to 8.9 ± 6.9 points. Details are shown in Table 5.

The rates of patients who died while on the waiting list and of those in too poor health for transplantation were comparable within their respective groups, according to their listing indication (see Table 1). 

## 4. Discussion

Our institution has shown that not only the MELD score itself has a significant impact on post-transplant outcome, but also dynamic changes in the score [9,11]. Patients with severe deterioration during the waiting time suffered from significantly poorer post-transplant survival [8].

Although deterioration in MELD was already cited in 2003 as a predictive factor for waiting list mortality, it faded into oblivion [12,13]. More recently, Tang et al. reported that a deterioration of 40% in MELD-Na over a 90-day period resulted in poor prognosis in a subset of HCC patients [14]. 

The main concept of Delta MELD is the identification of deteriorating patients on the liver transplant waiting list to avoid poor post-transplant outcomes. A temporarily “put on hold” strategy might allow stabilization of these patients during a presumable infection and enable MELD-stable candidates to achieve more beneficial transplantations [8,9]. In contrast, an MELD-Na score obtained at a certain time point only provides physicians with the current medical status, but does not reflect the underlying dynamic in the course of end-stage liver disease.

In this analysis of the UNOS database, Delta MELD was a significant predictor of waiting list outcome, comparable to European data reported by our department [8,9]. In our comprehensive risk model, the likelihood of death on the waiting list tripled with a Delta MELD-Na of 10 compared to 1. In accordance with our data, Xun et al. reported comparable results for Delta MELD in patients with hepatitis B acute-on-chronic failure (ACLF) [15,16]. Nevertheless, ACLF patients are challenging to define and only very few of these patients get listed for transplantation with high waiting list mortality at the end [17]. 

In contrast, patients who remained listed or were removed because they were deemed to be too healthy for transplantation showed very stable MELD trends or even improvement, compared to their competing waiting list candidates. Further, patients who finally underwent transplantation presented with only minimal deterioration during the waiting time.

Not only Delta MELD-Na had a severe impact on waiting list outcome, but also the maximal deterioration in MELD-Na between the lowest and highest collected values during the waiting time (Delta MELD max). Again, DOL patients and those too sick for transplantation showed the highest Delta MELD-Na max values during the waiting time, compared to patients with favorable outcomes (transplantation, removed due to good condition). 

This study was also able to address the issue of sampling time: For DOL patients and those in too poor health for transplantation, the MELD-Na max and MELD-Na Off were measured within a very short time period. In 51% of DOL patients, MELD-Na max and MELD-Na Off were found to be identical. Comparable findings were made for patients too sick for transplantation (49%). However, in patients who finally underwent transplantation, identical MELD-Na max and MELD-Na last were only observed in 37% of cases. Because of the assumption that deterioration of patients usually happens close to the delisting event, we were also interested in the impact of the last MELD-Na alteration and the MELD-Na alteration during the last 30 days (Delta “last 30”) on the waiting list in relation to the outcome. 

Delta MELD-Na last was significantly higher for DOL patients and for those in too poor health for transplantation. Nevertheless, it was lower than the overall Delta during the waiting time and the Delta max. This was probably due to the short interval between the two last MELD-Na recordings. Delta “last 30” had a similar impact as Delta last on waiting list outcome, but the values were two to three times higher.

In patients listed for less than 30 days, we found very little alteration during their short waiting time. These patients started with very high MELD-Na values, which resulted in short listing periods. They were either allocated for transplantation (65%), which supported the MELD philosophy, or they failed by death (12%) or delisting due to poor medical condition (11%).

In general, patients who died on the waiting list showed higher MELD-Na On values than patients with more favorable waiting list outcomes. Interestingly, patients removed from the waiting list due to poor medical condition showed MELD-Na On scores comparable to those of patients who underwent transplantation. Taking this into consideration, MELD Score alone is not a comprehensive tool to assess disease severity.

In contrast, patients who were removed from the waiting list because of being in too good medical condition already showed significantly lower MELD-Na On scores than the two other groups. It has yet to be discussed critically whether listing these patients for transplantation is medically sensible. Comparably, low MELD-Na On values were found for the group of patients still listed. 

MELD-Na Off clearly reflects the medically severe disease conditions of DOL patients or those removed from the waiting list. Both groups showed higher MELD-Na delisting scores (MELD-Na Off) than patients at the time of transplantation. 

Consecutively, higher Delta MELD-Na values during the waiting time were observed in these patient groups as well. 

As this study was retrospective, conclusions should be drawn with caution. In coherence with previous literature, we excluded all patients receiving standard exception points or status-1 patients from analysis in order to avoid confounding bias. 

## 5. Conclusions

Severe medical deterioration in liver transplant candidates during waiting time results in significantly poorer waiting list outcomes. Assessment of MELD-Na alterations proved to be a helpful tool for risk estimation in these patients and might improve organ allocation.

## Figures and Tables

**Table 1:** Listing indications of the study population according to outcome.

	DOL	Too Poor	Active	Too Good	TX	Other	ALL
Alcoholic	1323 (12.3)	1162 (10.8)	2251 (21.0)	398 (3.7)	4743 (44.2)	862 (8.0)	10,739 (100.0)
Biliary	329 (11.5)	262 (9.1)	584 (20.4)	66 (2.3)	1540 (53.7)	87 (3.0)	2868 (100.0)
Hepatitis	1455 (11.1)	1452 (11.1)	2330 (17.8)	337 (2.6)	6682 (51.0)	842 (6.4)	13,098 (100.0)
Metabolic	77 (10.8)	56 (7.9)	97 (13.7)	8 (1.1)	451 (63.4)	22 (3.1)	711 (100.0)
NASH	744 (13.6)	668 (12.2)	1160 (21.2)	98 (1.8)	2538 (46.4)	262 (4.8)	5470 (100.0)
Other	475 (12.1)	485 (12.4)	846 (21.6)	143 (3.7)	1762 (44.9)	209 (5.3)	3920 (100.0)
ALL	4403 (12.0)	4085 (11.1)	7268 (19.7)	1050 (2.9)	17,716 (48.1)	2284 (6.2)	36,806 (100.0)

DOL = died on list, too poor = removed from waiting list because of being in too poor medical condition, active = still actively listed for transplantation, too good = removed because of being in too good medical condition, TX = transplanted. Patient numbers (and percentages) are presented.

**Table 2:** **a**. Waiting time according to listing indications. **b**. Waiting time according to MELD-Na ON. **c**. Waiting time according to waiting list outcome.

**a.**	
**Indications**	**Waiting Time**
Alcoholic	152 (24:432)
Biliary	192 (53:492)
Hepatitis	183 (40:436)
Metabolic	90 (10:310)
NASH	168 (39:419)
Other	147 (24:453)
**b.**	
**MELD-Na ON**	**Waiting Time**
<15	310 (122:600)
15–20	270 (115:549)
21–28	103 (34:267)
30–34	17 (6:48)
35>	5 (2:12)
**c.**	
**Outcome**	**Waiting Time**
TX	90 (16:260)
Too sick	144 (34:363)
DOL	123 (28:344)
Active	469 (217:908)
Too good	531 (277:868)
Other	256 (91:513)

Kruskal–Wallis test: *p* < 0.001. Waiting time in days presented in median values (1st quartile: 3rd quartile). DOL = died on list, too sick = removed from waiting list because of being in too poor medical condition, active = still actively listed for transplantation, too good = removed because of being in too good medical condition, TX = transplanted.

**Table 3:** MELD-Na assessments according to waiting list outcome.

	Number of Patients	MELD-Na ON	MELD-Na OFF	Delta	Delta Max	Delta Last
TX	17,716	21.6 ± 9.1	24.6 ± 9.8	3.0 ± 6.3	6.6 ± 6.1	−0.3 ± 3.9
Too sick	4085	21.5 ± 8.5	27.6 ± 10.1	6.1 ± 8.0	9.3 ± 7.3	2.0 ± 5.7
DOL	4403	22.5 ± 8.0	29.3 ± 9.1	6.8 ± 8.4	10.0 ± 7.6	2.8 ± 6.4
active	7268	15.4 ± 5.2	15.3 ± 5.7	−0.1 ± 5.2	6.7 ± 5.1	−0.2 ± 3.5
Too good	1050	15.5 ± 6.3	12.3 ± 4.7	−3.4 ± 5.8	6.7 ± 5.5	−1.1 ± 3.9
Other	2284	16.9 ± 6.5	17.5 ± 7.3	0.6 ± 5.6	5.9 ± 5.1	0.5 ± 4.3
All	36,806	20.0 ± 8.5	22.9 ± 10.2	2.8 ± 7.0	7.3 ± 6.3	0.4 ± 4.6
		**MELD-Na ON**	**MELD-Na OFF**	**Delta**	**Delta Max**	**Delta Last**
Too sick		0.2	<0.01	<0.01	<0.01	<0.01
DOL		<0.01	<0.01	<0.01	<0.01	<0.01
Too good		<0.01	<0.01	<0.01	0.8	<0.01

Meld-Na ON = listing MELD-Na, MELD-Na OFF = MELD-Na at delisting, Delta = difference between MELD-Na Off and MELD-Na On, Delta max = maximal difference between the lowest and the highest MELD-Na measurements during waiting time, Delta last = MELD-Na OFF minus the next-to-last available MELD-Na score. Values are presented as mean ± standard deviation. *p*-values in relation to finally transplanted patients (*t*-test).

**Table 4:** MELD-Na assessments for patients listed <30 days.

	Number of Patients	MELD ON	MELD OFF	Delta	Delta Max
TX	5744	29.3 (8.8)	30.1 (9.0)	0.7 (4.3)	3.4(3.8)
Too sick	961	29.4 (9.5)	31.8 (10.0)	2.5 (5.2)	4.0 (4.8)
DOL	1117	29.7 (8.6)	31.8 (9.1)	2.1 (4.8)	3.6 (4.6)
Too good	62	18.1 (9.7)	16.2 (8.2)	−1.9 (4.7)	2.9 (5.0)
active	716	12.1 (4.7)	12.1 (4.7)	0.0 (0.9)	0.1 (0.9)
other	313	18.5 (9.0)	18.5 (8.9)	0.0 (2.4)	1.3 (2.5)
All	8913	27.5 (10.0)	28.5 (10.4)	1.0 (4.3)	3.1 (4.0)
		**MELD-Na ON**	**MELD-Na OFF**	**Delta**	**Delta Max**
Too sick		1	<0.01	<0.01	<0.01
DOL		0.2	<0.01	0.2	<0.01

Values are presented as mean and standard deviation). DOL = died on list, too sick = removed from waiting list because of too poor medical condition, TX = transplanted. *p*-values in relation to finally transplanted patients (*t*-test).

**Table 5:** MELD-Na assessments depending on listing indication.

	MELD ON	MELD OFF	Delta	Delta Max	Delta Last
Alcoholic	22.1 (8.5)	23.8 (10.0)	1.7 (6.8)	7.1 (5.9)	0.1 (4.5)
Biliary	19.2 (7.9)	23.5 (10.0)	4.3 (7.6)	8.9 (6.9)	0.2 (4.8)
Hepatitis	18.1 (8.3)	21.0 (10.4)	3.0 (6.9)	6.8 (6.4)	0.6 (4.6)
Metabolic	22.0 (9.1)	25.6 (7.1)	3.5 (7.1)	7.2 (6.5)	0.1 (4.7)
NASH	20.0 (7.7)	23.8 (9.5)	3.8 (7.0)	7.9 (6.4)	0.5 (4.6)
Other	21.2 (8.5)	24.2 (10.1)	3.0 (7.3)	7.8 (6.6)	0.3 (4.5)
**MELD-Na ON**		**MELD-Na OFF**	**Delta**	**Delta Max**	**Delta Last**
<0.01		<0.01	<0.01	<0.01	<0.01

Values are presented as mean and (standard deviation). ANOVA Test.

## Data Availability

The data that support the findings of this study are available from OPTN. Restrictions apply to the availability of these data, which were used under license for this study. Data are available from the authors with the permission of OPTN. OPTN has approved this research and the use of OPTN data.

## Abbreviations

MELD	model for end-stage liver disease
Delta MELD	dynamic MELD deterioration
Na	Sodium
TX	Transplantation
DOL	Died on list
UNOS	United Network of Organ Sharing
